# Targeting Neuroinflammation in Brain Cancer: Uncovering Mechanisms, Pharmacological Targets, and Neuropharmaceutical Developments

**DOI:** 10.3389/fphar.2021.680021

**Published:** 2021-05-18

**Authors:** Mahmoud S. Alghamri, Brandon L. McClellan, Carson S. Hartlage, Santiago Haase, Syed Mohd Faisal, Rohit Thalla, Ali Dabaja, Kaushik Banerjee, Stephen V. Carney, Anzar A. Mujeeb, Michael R. Olin, James J. Moon, Anna Schwendeman, Pedro R. Lowenstein, Maria G. Castro

**Affiliations:** ^1^ Department of Neurosurgery, University of Michigan Medical School, Ann Arbor, MI, United States; ^2^ Department of Cell and Developmental Biology, University of Michigan Medical School, Ann Arbor, MI, United States; ^3^ Department of Pediatrics, University of Minnesota, Minneapolis, MN, United States; ^4^ Masonic Cancer Center, University of Minnesota, Minneapolis, MN, United States; ^5^ Department of Pharmaceutical Sciences, University of Michigan, Ann Arbor, MI, United States; ^6^ Biointerfaces Institute, University of Michigan, Ann Arbor, MI, United States; ^7^ Department of Biomedical Engineering, University of Michigan, Ann Arbor, MI, United States; ^8^ Rogel Cancer Center, University of Michigan Medical School, Ann Arbor, MI, United States; ^9^ Biosciences Initiative in Brain Cancer, University of Michigan, Ann Arbor, MI, United States

**Keywords:** immunosuppression, inflammation, tumor microenvironment, glioma, immunotherapy

## Abstract

Gliomas are one of the most lethal types of cancers accounting for ∼80% of all central nervous system (CNS) primary malignancies. Among gliomas, glioblastomas (GBM) are the most aggressive, characterized by a median patient survival of fewer than 15  months. Recent molecular characterization studies uncovered the genetic signatures and methylation status of gliomas and correlate these with clinical prognosis. The most relevant molecular characteristics for the new glioma classification are *IDH* mutation, chromosome 1p/19q deletion, histone mutations, and other genetic parameters such as *ATRX* loss, *TP53,* and *TERT* mutations, as well as DNA methylation levels. Similar to other solid tumors, glioma progression is impacted by the complex interactions between the tumor cells and immune cells within the tumor microenvironment. The immune system’s response to cancer can impact the glioma’s survival, proliferation, and invasiveness. Salient characteristics of gliomas include enhanced vascularization, stimulation of a hypoxic tumor microenvironment, increased oxidative stress, and an immune suppressive milieu. These processes promote the neuro-inflammatory tumor microenvironment which can lead to the loss of blood-brain barrier (BBB) integrity. The consequences of a compromised BBB are deleteriously exposing the brain to potentially harmful concentrations of substances from the peripheral circulation, adversely affecting neuronal signaling, and abnormal immune cell infiltration; all of which can lead to disruption of brain homeostasis. In this review, we first describe the unique features of inflammation in CNS tumors. We then discuss the mechanisms of tumor-initiating neuro-inflammatory microenvironment and its impact on tumor invasion and progression. Finally, we also discuss potential pharmacological interventions that can be used to target neuro-inflammation in gliomas.

## Introduction

Gliomas are the most commonly diagnosed malignant primary tumors that arise in the brain (Louis et al., 2016; Ceccarelli et al., 2016; Schwartzbaum et al., 2006). The traditional classification of gliomas is based on the histological features according to the microscopic similarity with the putative cell of origin along glial precursor cell lineages (i.e., astrocytes, oligodendrocytes, etc) and malignancy grade (Grade I-IV). Gliomas that lack features of aggressiveness and grow slower are classified as Low-Grade Gliomas (LGG) and correspond with WHO grades I and II, while gliomas that have hallmarks of aggressiveness and are more proliferative are classified as High-Grade Gliomas (HGG) and correspond with WHO grades III and IV. Although histopathologic classification is more established, it has the disadvantage of potential observer bias, particularly between grade III and IV tumors (van den Bent et al., 2010). In the last decade, the availability of glioma datasets has facilitated the correlation of genetic, transcriptional, and epigenetic signatures with clinical features (Noushmehr et al., 2010; Sturm et al., 2012; Verhaak et al., 2010).

The current stratification of gliomas is established by the WHO Classification of Central Nervous System Tumors, which was updated in 2016 (Louis et al., 2016; Wesseling and Capper, 2018). According to the new classification, gliomas are divided into diffusely infiltrating gliomas, which include grade II and III astrocytic tumors, grade II and III oligodendrogliomas, grade IV glioblastoma, and diffuse midline gliomas (Louis et al., 2016). This recent update is the first to include molecular parameters into consideration, besides the commonly used histopathological parameters. The more relevant molecular markers incorporated in glioma classification are *IDH* mutations, 1p19q deletion, MGMT promoter methylation, TERT promoter mutations, ATRX loss of function mutations, and p53 loss of function mutations and mutations in isocitrate dehydrogenase 1 and 2 genes (*IDH1/*2 m) (Louis et al., 2016).

*IDH1/2* m defines a distinct subgroup of glioma (GBM) and is clinically associated with favorable outcomes. *IDH*m have been identified in grade II and III astrocytomas, oligodendrogliomas, and oligoastrocytomas, and in secondary glioblastomas (which refers to gliomas with high-grade hallmarks such as nuclear atypia, and/or cellular pleomorphism as well as microvascular proliferation and/or necrosis), which result from tumor recurrence. *IDH1*m tumors are classified into two molecular subgroups: the first group carries a codeletion of the chromosomal bands 1p and 19q and TERT promoter mutation. Most of these gliomas are histologically defined as oligodendrogliomas. On the other hand, *IDH*m without 1p and 19q codeletion are mostly P53 and ATRX mutant, associated with hypermethylation phenotype (G-CIMP high) and astrocytic histology. The incorporation of *IDH*m status into diffuse glioma classification was in part prompted by its relevance in the clinical outcome of the tumors.

Diffuse gliomas with wild-type *IDH* (*IDH*-wt), even when they may be histologically defined as grade II or III, tend to behave like more aggressive glioblastomas. *IDH*-wt grade IV glioblastomas (GBM) are the most common malignant primary brain tumors. GBMs are densely cellular, pleomorphic tumors with mitotic activity and either microvascular proliferation or necrosis, or both. Histologic variants of this group include epithelioid glioblastoma (which normally occurs in children and young adults and carries BRAF V600 E mutations), along with giant cell GBM and gliosarcoma. The prognosis for all glioblastoma variants is poor, with survival commonly less than two years. Despite the clear differences between them, pediatric gliomas are still classified according to the histological resemblance to adult gliomas. As an exception, the WHO 2016 classification incorporated the entity of histone H3 K27 M diffuse midline glioma, which mainly occurs in pediatric patients and regardless of the histological grade, has a poor prognosis. Our molecular knowledge of pediatric gliomas has been refined over the last few years, and some molecular markers, such as H3 G34R/V, BRAF, NF1 mutations, and PDGFRα amplifications, are now becoming relevant for the decision of clinical interventions (Koschmann et al., 2016; Miklja et al., 2019; Haase et al., 2020).

Several aspects make it difficult to efficiently treat gliomas, i.e., the anatomical location, the presence of the blood-brain barrier (BBB), and the restricted immune reactivity within the CNS. The anatomical location, together with the infiltrative nature of high-grade glioma, makes the total resection of the tumor mass virtually impossible. The blood-brain barrier hampers the delivery of therapeutic compounds to the tumor site. Despite these limitations, intense research has opened up opportunities to explore tailored therapies for different glioma subtypes with particular molecular lesions that are currently under clinical trials (Haase et al., 2020).

Currently, the standard of care (SOC) for GBM is comprised of surgery to remove the tumor mass, followed by radiation therapy in combination with Temozolomide (TMZ) administration. The incorporation of TMZ in GBM treatment leads to a small progression-free survival (PFS) improvement (5 vs. 6.9 months), in addition to a significant benefit in 2-years overall survival (11 vs. 27%) (Brandes et al., 2008). The MGMT promoter methylation can predict the TMZ therapy efficacy, and in many glioma subtypes which do not have MGMT promoter methylation, TMZ does not improve patients’ outcome. The extent of tumor resection is the most prominent treatment factor associated with survival (Brown et al., 2016; Molinaro et al., 2019).

## Blood-Brain Barrier

**FIGURE 1:**
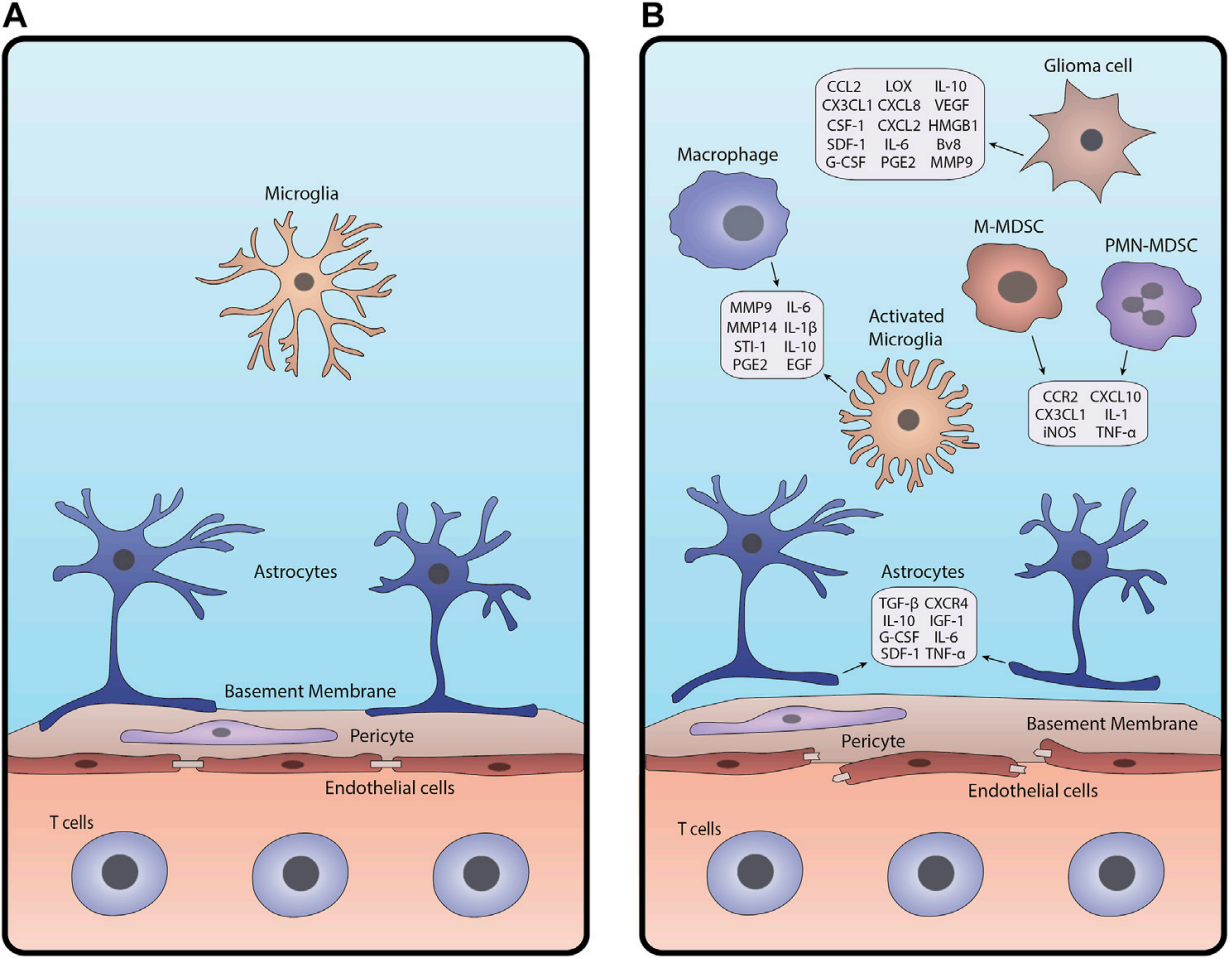
Schematic of the BBB under **(A)** homeostasis and **(B)** glioma-associated inflammation. In inflamed conditions **(B)**, junctions between endothelial cells break apart, pericytes are disrupted, and astrocytic endfeet detach from the basement membrane. Glioma cells, macrophages and microglia, astrocytes, and MDSCs release cytokines and other factors that contribute to BBB breakdown and create an inflammatory tumor microenvironment.

The healthy Blood-Brain Barrier (BBB) is a selectively permeable barrier secured by endothelial cells linked by tight junctions, pericytes embedded in a basement membrane. Astrocytic end-feet is also anchored to the basement membrane (Varatharaj and Galea, 2017; Ros et al., 2018). Under homeostasis, these components protect the brain from toxic materials, regulate the transport of essential nutrients, and maintain a stable brain environment (Bergers and Benjamin, 2003; Ros et al., 2018). When BBB integrity is compromised, for example by glioma-associated inflammation, these cell layers change in both anatomy and function, altering the permeability of blood vessels in the brain (Figure 1; Erickson et al., 2012; Varatharaj and Galea, 2017).

Gliomas are characterized by the activation of numerous pathways that drive gliomagenesis. These include but are not limited to activation of hypoxia, abnormal angiogenesis, and tissue remodeling which disrupt the BBB and trigger an inflammatory response in the brain microenvironment (Bergers and Benjamin, 2003; Allavena et al., 2008; Ros et al., 2018). Response to hypoxic conditions is mediated by hypoxia-inducible factor-alpha (HIF-α), regulating the expression of angiogenic and inflammatory factors such as vascular endothelial growth factor (VEGF) (Murat et al., 2009; Belykh et al., 2020). VEGF disrupts the cellular barrier around existing blood vessels, pulling endothelial cells away to form new capillaries with fenestrations and fewer tight junctions (Dubois et al., 2014; Belykh et al., 2020). This results in an enhanced infiltration of cellular and plasma components into the brain, further exacerbating brain homeostasis.

While the intact BBB permits immune surveillance by effector immune cells, compromised BBB allows excessive immune cell infiltration magnifying inflammatory responses (Vries et al., 1998; Sonar and Lal, 2018). Tumor necrosis factor-alpha (TNF-α) and interleukin-1 beta (IL-1β) produced by inflamed immune cells such as tumor-associated macrophages (TAMs) promote the expression of inflammatory molecules in other cell types (Allavena et al., 2008; Sethi et al., 2008; Kore and Abraham, 2014). Moreover, reactive astrocytes, microglia, and endothelial cells can secrete mediators including transforming growth factor (TGF-β) and metalloproteinases (MMP2 and MMP9), augmenting the inflammatory tumor microenvironment (Sethi et al., 2008; Könnecke and Bechmann, 2013). Altered TGF-β signaling reduces the expression of cell adhesion molecules in pericytes and endothelial cells, weakening their attachments to the surrounding vessel (Joseph et al., 2013; Sonar and Lal, 2018). MMPs secreted by activated microglia cleave extracellular (ECM) components of the basal lamina, which alters the function of the basement membrane (Wolburg et al., 2012; Könnecke and Bechmann, 2013).

## Glioma Tumor Microenvironment

**FIGURE 2:**
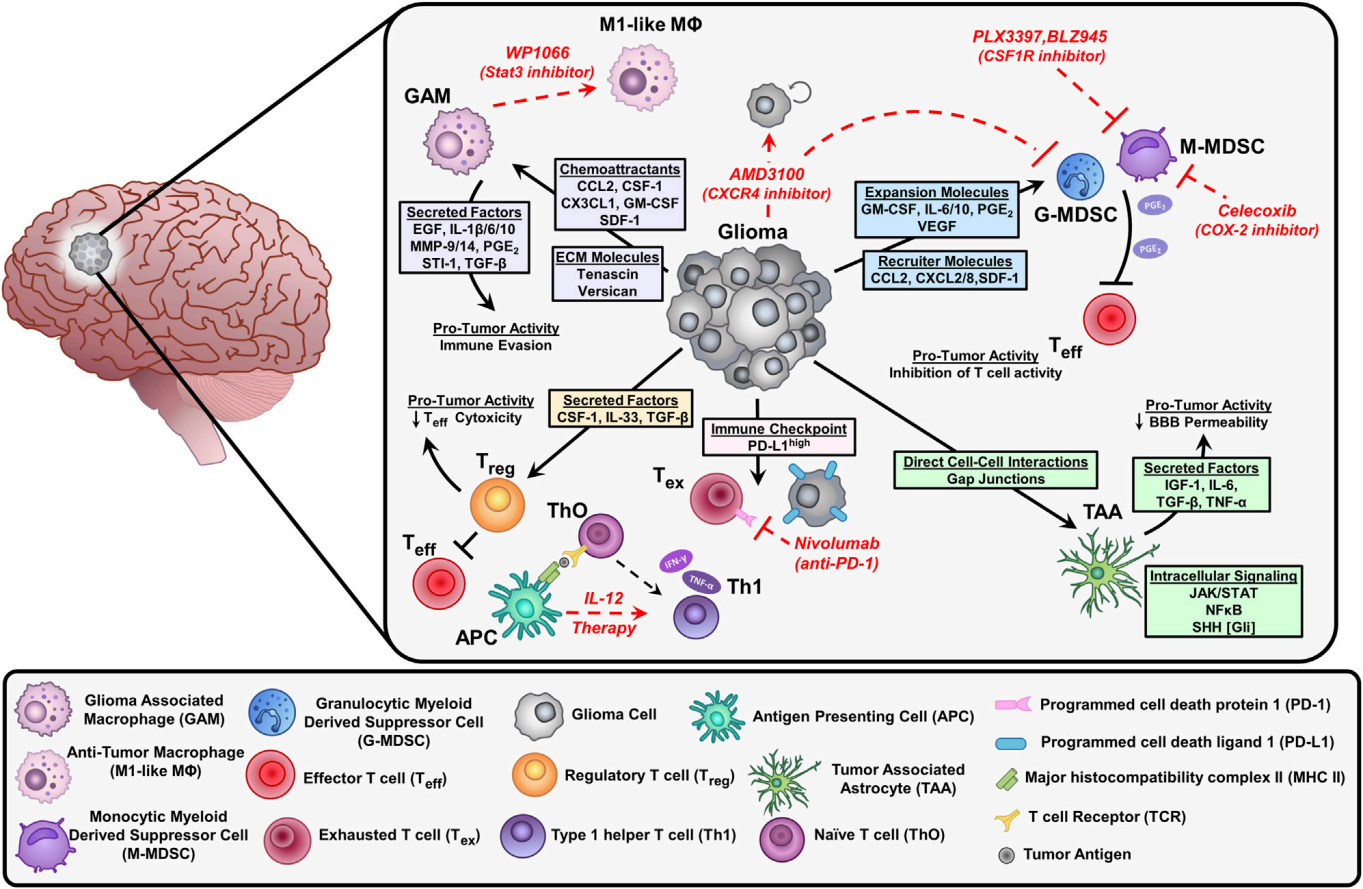
Depiction of the immunosuppressive environment in glioblastoma and current therapeutic approaches (red) to stimulate anti-tumor responses or inhibit suppressive immune cell populations. Glioma cells release chemoattractants and cytokines that recruit immune cells into the tumor microenvironment (TME). These factors elicit pro-tumor activities that inhibit effector T cells (Teff) and contribute to tumor progression. Glioma cells can also directly interact with immune cells and astrocytes impacting the effectiveness of chemotherapy. Inhibition of Stat3 by WP1066 reduces glioma-associated macrophages (GAM) by blocking intracellular signals induced by IL-10 to promote an anti-tumor macrophage (M1-like MΦ) phenotype. The CXCR4 antagonist AMD3100 prevents the binding of stromal cell-derived factor (SDF-1), resulting in decreased tumor proliferation and blocks the migration of myeloid-derived suppressor cells (MDSCs) in the TME. The CSF1R inhibitors (PLX3397, BLZ945) suppress the infiltration of myeloid cells (macrophages and MDSCs) within the TME. MDSCs synthesize prostaglandin E2 (PGE2) via COX-2 which inhibits effector T cells by reducing Interferon-gamma (IFN-γ) release. The COX-2 inhibitor, celecoxib reduces MDSC immunosuppressive activity. Interleukin-12 (IL-12) is secreted by antigen-presenting cells (APC) like dendritic cells and macrophages, exogenous IL-12 therapy triggers a pro-inflammatory transition of naïve T cells (ThO) to type 1 helper T cells (Th1) leading to an increase in IFN-y and tumor necrosis factor (TNF-α) secretion. Tumor cells express high levels of programmed cell death ligand (PD-L1) on their cell surface, which leads to exhausted T cells (Tex) by immune checkpoint signaling through their receptor programmed cell death protein 1 (PD-1). Inhibition of PD-1 with nivolumab reduces T cell exhaustion.

Previously, the CNS was thought to be an ‘immune privileged’ site. This idea was favored following the rejection of transferred foreign tissue into the brain parenchyma (Murphy and Sturm, 1923; Medawar, 1948; Galea et al., 2007). Further investigation demonstrated that this immune privilege status of the CNS is relative to other tissues and organs and it depends on the existence of neuroinflammation. This has led to the implementation of clinical trials aiming to develop immunotherapies against glioma. However, most of these trials have failed to show benefits in glioma patients due to the immunosuppressive nature of the glioma tumor microenvironment (TME) that hampers effective anti-glioma immune response. In addition to the infiltrating immune cells, glioma cells express immune suppressive molecules such as interleukin-6, interleukin-10, TGF-β, LDH5, galectin-1 (gal-1), and prostaglandin-E which reprogram the infiltrating immune cells to a pro-tumor phenotype (Figure 2; Van Meir, 1995; Huettner et al., 1997; Crane et al., 2012; Perng and Lim, 2015). Interleukin-10 not only inhibits the functions of DCs and macrophages but also has a pro-inflammatory and inhibitory effect against T-cell activation and proliferation (Moore et al., 2001). Gal-1 enhances tumor cell migration and induces T-cell apoptosis and the skewing of tumor-infiltrating macrophages to the immunosuppressive M2 type (Verschuere et al., 2011).

Myeloid cells are the most frequent immune cell type in the glioma TME, accounting for ∼60% of all glioma infiltrating immune cells. In GBM, myeloid-derived suppressor cells (MDSCs) are major immunosuppressive cells that hamper glioma immune response (Kamran et al., 2017; Alghamri et al., 2020). Clinically, the frequency of MDSCs is associated with an unfavorable prognosis in glioma (Rahbar et al., 2016). Besides MDSCs, glioma-associated macrophages/microglia (GAMs) represent a major population of immune cells infiltrating gliomas (Pyonteck et al., 2013; Brown et al., 2018; Chen and Hambardzumyan, 2018). Several chemokines and cytokines, including CCL2 and CX3CL1, are responsible for GAMs recruitments and expansion in glioma (Okada et al., 2009; Held-Feindt et al., 2010; da Fonseca and Badie, 2013). Within the lymphoid cells, T cells and NK cells represent the major effector cells in glioma (Kmiecik et al., 2013; Kmiecik et al., 2014), albeit they represent a small percentage of the total immune cells within the glioma TME (about 2% of immune-infiltrating cells). Tregs are also infiltrated in the glioma TME, they elicit a strong immunosuppressive response against anti-glioma T-cells (Chang et al., 2016). Multiple GBM-derived factors are responsible for Tregs recruitment including CCL22, CCL2, TGF-β, or indoleamine 2,3-dioxygenase 1 (IDO1) (Akasaki et al., 2004; Akhurst and Hata, 2012; Wainwright et al., 2012; Chang et al., 2016). Glioma-induced tissue hypoxia has also been shown to be responsible for the activation of regulatory T-cells (Saetta et al., 2011; Razavi et al., 2016). Collectively, recruitment and activation of immunosuppressive cells decrease the efficacy of anti-glioma immune response. Below, we will discuss in detail how some of these cells are recruited and activated to the glioma TME to support glioma invasion.

### Role of Myeloid Cells (Microglia/Macrophages, MDSCs)

GAMs make up a high proportion of myeloid cells encountered in the glioblastoma microenvironment accounting for up to 30% of the tumor mass (Russo and Cappoli, 2018). Microglia are the brain’s resident myeloid cells, which are not replenished by blood-derived monocytes under normal physiological conditions. Instead, they function as a self-sustaining population with an extended capacity to proliferate (Schetters et al., 2018). In contrast, peripheral macrophages are derived from hematopoietic stem cells arising in the bone marrow and migrate to the glioma microenvironment partially due to the breakdown of the blood-brain barrier (Russo and Cappoli, 2018). GAMs are recruited to the glioma microenvironment via the glioma cells’ release of chemoattractant: CCL2, CX3CL1, colony-stimulating factor (CSF-1), stromal cell-derived factor 1 (SDF-1), granulocyte/macrophage-colony stimulating factor (GM-CSF), and lysine oxidase (LOX) (Figure 2; Zhang et al., 2012; Roesch et al., 2018; Gutmann and Kettenmann, 2019; Chen et al., 2019). Under pathological conditions, microglia and peripheral macrophages/monocytes activate, proliferate, and contribute to the disruption of immunological homeostasis (Gutmann and Kettenmann, 2019; Sankowski et al., 2019). In the glioma microenvironment, macrophages are impacted to elicit pro-tumoral effects through the activation of immunosuppressive pathways enhancing glioma progression (Hambardzumyan et al., 2016; Roesch et al., 2018). For example, glioma cells produce let7, tenascin-C (TNC), and versican resulting in increased production of cytokines and matrix metalloproteases (MMP9 and MMP14) by GAMs (Hambardzumyan et al., 2016; Gutmann and Kettenmann, 2019). In response to these signals, GAMs release multiple cytokines such as transforming growth factor *ß* (TGF-β), stress-inducible protein 1 (STI-1), prostaglandin E2 (PGE_2_), IL-6, IL-1β, IL-10, and epidermal growth factor (EGF), which promote glioma cell proliferation and inhibit T cells function (Hambardzumyan et al., 2016; Gutmann and Kettenmann, 2019). GAMs’ depletion and/or inhibition by chlodronate and microglial inhibitory factor (MIF/TKP) drastically reduced tumor growth, further suggesting GAMs as a potential therapeutic target (Markovic et al., 2009; Zhai et al., 2011).

Myeloid-derived suppressor cells (MDSCs) are a heterogeneous population of immature myeloid cells that express high levels of immunosuppressive molecules and inhibit anti-tumor immunity (Grabowski et al., 2021). These cells can derive from monocytic (M-MDSCs) or granulocytic (PMN-MDSCs) origin (Grabowski et al., 2021). M-MDSCs have been shown to have greater immunosuppressive capability and are more common in the blood of GBM patients; whereas, PMN-MDSCs make up a greater portion of MDSCs in the glioma microenvironment (Mi et al., 2020). Tumor-derived cytokines are the major drivers of MDSCs expansion in the glioma microenvironment. These can be divided into two classes: MDSCs recruiters (such as CCL2, CXCL8, SDF-1, and CXCL2) and MDSCs expanders (such as IL-6, PGE2, IL-10, VEGF, and GM-CSF) (Mi et al., 2020; Miyazaki et al., 2020). These cytokines result in the recruitment and expansion of MDSCs infiltrating the glioma microenvironment. There, MDSCs suppress mainly T cell and NK cell functions (Gieryng et al., 2017). This inhibition is triggered by multiple mechanisms including induction of oxidative stress, inhibition of T cell migration, expression of T cell inhibitory ligands, and depletion of critical T cell metabolites (Mi et al., 2020; Grabowski et al., 2021). The establishment of MDSCs as a major immunosuppressive population identifies them as a target for anti-glioma therapy.

Several immunotherapies are being evaluated in clinical trials to target the immunosuppressive and pro-tumoral myeloid cells. The large majority of these immunotherapies target the pro-tumoral myeloid cell recruitment to the glioma. Representatively, the chemoattractant molecules responsible for the myeloid cell migration to the glioma, for example, CSF-1R, avβ3/5 integrins, and CXCR4 are being targeted in multiple trials (Russo and Cappoli, 2018; Roesch et al., 2018). PLX3397 (ClinicalTrials.gov NCT identifiers: CNCT01349036 and NCT01790503) and BLZ945 (ClinicalTrials.gov NCT identifier: NCT02829723) are CSF-1R inhibitors currently being evaluated for their efficacy as glioblastoma treatments (Butowski et al., 2015; Colman et al., 2018). Unfortunately, patients treated with PLX3397 alone showed no significant improvement, nor did PLX3397 improve the results when combined with the current standard of care (SOC), temozolomide chemoradiotherapy. The trial utilizing BLZ945, alone and in combination with the PD-1 checkpoint receptor inhibitor PDR001, as a treatment of glioblastoma, is still ongoing. An inhibitor of avβ3 and avβ5 integrins, cilengitide, in combination with SOC has been evaluated in clinical trials (ClinicalTrials.gov NCT identifier: NCT00689221) and shown no survival benefits compared to SOC alone. CXCR4 inhibitor, AMD3100, is in clinical trials for treating GBM, and one completed trial of AMD3100 in combination with SOC (ClinicalTrials.gov NCT identifier: NCT01977677) showed an improvement in tumor control (Thomas et al., 2019). In addition to inhibiting the recruitment of myeloid cells, some immunotherapy agents are also being evaluated in trials to target the functions of GAMs and MDSCs (Roesch et al., 2018; Mi et al., 2020). For stimulating GAM anti-tumoral activity, the small molecule inhibitor of STAT3, WP1066, is being evaluated in clinical trials (ClinicalTrials.gov NCT identifiers: NCT01904123 and NCT04334863). Research has shown that WP1066 causes GAM upregulation of costimulatory molecules CD80 and CD86, but no results have been released for the WP1066 clinical trials (Hussain et al., 2007). Studies have shown that COX-2 inhibition suppresses MDSC activation, however COX-2 inhibition, via celecoxib, failed to show any favorable outcome in clinical settings (ClinicalTrials.gov NCT identifier: NCT00112502) (Penas-Prado et al., 2015; Mi et al., 2020).

### Role of T-Cells

T cells are the largest group of lymphocytes infiltrating the glioma microenvironment and are key factors for effective anti-glioma immunity (Yang et al., 2010; Mohme and Neidert, 2020). The number of CD8 T cells infiltrating the glioma microenvironment is correlated with positive outcomes in clinical settings (Yang et al., 2010; Gieryng et al., 2017). The first step for initiation and development of anti-glioma CD8^+^ T cells, i.e., T cell priming, takes place in lymphoid tissues (cervical draining lymph nodes). The sequence of events begins with antigen loading on DCs and culminates with CD8^+^ T cell‐mediated tumor cytolysis (Dranoff, 2004; Farhood et al., 2019). DCs cross-present the tumor-associated antigen via MHC class I molecules to CD8^+^ T cells to trigger the formation of cytotoxic anti-glioma CD8^+^ T cells (CTLs) (Joffre et al., 2012; Spranger and Gajewski, 2018). The activated CTLs use two main pathways to induce target cell apoptosis, i.e., granule exocytosis and Fas ligand (FasL)‐mediated apoptosis (Voskoboinik et al., 2006; Anel et al., 1994; de Saint Basile et al., 2010; Hassin et al., 2011). The former is mediated by releasing granular proteins such as granzymes (Gzs)and perforin from the CTLs. The second mechanism is triggered by the FasL pathway which induces apoptosis through activation of caspases. This prompts the release of cytochrome *c* and other essential enzymes in the target cells. Other effector molecules being releases by the CTLs, such as interferon‐γ (IFN‐γ) and tumor necrosis factor *α* (TNF‐α), augment their cytotoxicity toward cancer cells [(Schoenborn and Wilson, 2007; Farhood et al., 2019)].

However, studies found that the majority of CD8^+^ T cells infiltrating the glioma microenvironment are in an exhausted, hypo-responsive state (Woroniecka et al., 2018). Exhaustion is a hypo-responsive T-cell state resulting from chronic antigenic exposure under sub-optimal conditions. This state represents a specific transcriptional program that is often characterized by up-regulation of various co-inhibitory receptors including PD-1, LAG-3, and TIM-3, on the T cells (Koyama et al., 2016; Kadiyala et al., 2020; Mohme and Neidert, 2020). These exhausted CD8^+^ T cells (CD8^+^ T_ex_ cells) have lower secretion of their effector cytokines, but they still provide some, albeit reduced antitumor immunity (Woroniecka et al., 2018).

On the other hand, CD4^+^ T cells primarily mediate anti-tumor immunity by providing help for CD8^+^ CTL and antibody responses, as well as via secretion of effector cytokines such as interferon-γ (IFNγ) and tumor necrosis factor-α (TNFα). CD4^+^ T cells are highly versatile, polyfunctional cells that can differentiate into one of several diverse functional subtypes (Tay et al., 2021). The most extensively studies subtype in glioma immunology is Tregs. While exhausted CD8 T cells are merely non-functional, Tregs acquire immunosuppressive function and have pro-tumor functions within the glioma microenvironment (Andaloussi and Lesniak, 2006; Mu et al., 2017). Tregs suppress the anti-tumor immune response directly via expression of immunosuppression inducing ligands, such as PD-L1 and CTLA-4, or indirectly via the release of anti-inflammatory cytokines, including IL-10 and TGF-β (Grabowski et al., 2021). Therefore, Tregs represent an attractive target to enhance glioma immunotherapy.

A variety of therapeutic approaches targeting the T cell compartment in gliomas are being evaluated for clinical testing. Monoclonal antibody-based therapies targeting the immune checkpoint molecules mainly PD-1, PD-L1, and CTLA-4 are being used to block the direct cell-to-cell contact-mediated inhibition of CD8^+^ T cells (Domingues et al., 2016; Wang et al., 2019). So far, clinical trials using the immune checkpoint inhibitors separately have shown inconsistent results in improving patient survival (Wang et al., 2019). For example, CheckMate 143 (ClinicalTrials.gov NCT identifier: NCT02017717) investigated the use of nivolumab, an anti-PD-1 monoclonal antibody, on patients with recurrent GBM, in a phase III clinical trial, but the trial was ended due to failure to improve overall survival compared to bevacizumab, an anti-VEGF therapy (Mitchell and Dey, 2019). However, this study prompted further investigation by showing the efficacy of nivolumab on the overall survival of a subset of recurrent GBM patients (Mitchell and Dey, 2019). A later phase II clinical trial (ClinicalTrials.gov NCT identifier: NCT02550249) demonstrated the safety of presurgical (neoadjuvant) nivolumab in a set of primary and recurrent GBM patients. Although no obvious clinical benefit was observed in recurrent GBM patients, 2 of the 3 primary patients treated with neoadjuvant nivolumab showed long-term survival (Schalper et al., 2019). Potential reasons for this lack of efficacy are low infiltration of CD8^+^ T cells in the tumor and several immune checkpoint molecules being expressed simultaneously. Several combination therapies are being tested to counteract these reasons for low efficacy encountered when using single-treatment modalities. These combination therapies utilize multiple checkpoint inhibitors along with DC-based vaccines to stimulate more CD8^+^ T cell recruitment (Wang et al., 2019; Miyazaki et al., 2020). ClinicalTrials.gov NCT identifier: NCT02529072 is an example of a recently tested DC-based vaccine combined with the nivolumab on recurrent gliomas. This study was terminated early due to adverse clinical effects, inefficacy of the combination and nivolumab alone treatments, and the failure of nivolumab alone to improve GBM survival (Peters et al., 2019). The combination immune checkpoint inhibitor therapies haven’t shown consistent efficacy yet, but more research has the potential to optimize the immune checkpoint inhibitor combinations to enhance clinical outcomes (Wang et al., 2019).

### Role of Astrocytes

The brain microenvironment consists of multiple cell types, including the most abundant glial cell, astrocytes. Astrocytes constitute the majority of brain cells ∼50% of all brain cells and, therefore, they are expected to play a major role in GBM [(O'Brien et al., 2013; Gieryng et al., 2017)]. GBM cells activate tumor-associated astrocytes (TAAs) to enhance GBM invasion within healthy brain tissue (Guan et al., 2018; Henrik Heiland et al., 2019). TAAs are also involved in tumor progression and resistance to radiation and chemotherapy (Yang et al., 2014). Astrocytes themselves can become tumorigenic, which can be triggered by aberrant signaling in essential genes related to astrocyte development. For example, the nuclear factor kappa‐B (NF‐κB)‐ pathway has been shown to be involved in the transformation of normal astrocytes into TAAs which enhance GBM evasion (Figure 2; Kim et al., 2014). Recently, STAT‐3 was found to play a major role in the induction of reactive astrocytes that contribute to the metastatic melanoma to the brain (Priego et al., 2018). Moreover, astrocytes secrete factors that maintain the tight junctions of the blood-brain barrier (BBB) (Abbott et al., 2006; Alvarez et al., 2011; Heithoff et al., 2021), which in turn regulates the success or failure of metastatic cells extravasating the brain. Tumor cells within the brain make direct contact with astrocytes through gap junctions (Soroceanu et al., 2001; Horng et al., 2017), leading to increased chemoresistance (Chen et al., 2015). Astrocytes also promote the release of degradative enzymes, cytokines, chemokines, and growth factors, thereby promoting tumor cell proliferation, survival, and invasion (Brandao et al., 2019).

Distinct astrocytic phenotype exists in the tumor environment, which leads to a large release of anti-inflammatory cytokines such as TGFβ, IL10, and G-CSF through JAK/STAT pathway activation (Henrik Heiland et al., 2019). Inhibition of the JAK/STAT pathway shifts the balance of pro- and anti-inflammatory cytokines toward a pro-inflammatory environment. The complex interaction of astrocytes and microglial cells can further contribute to the immunosuppressive glioma TME, suggesting that tumor-associated astrocytes contribute to anti-inflammatory responses (Henrik Heiland et al., 2019). Studies show that astrocytes with activated sonic hedgehog (SHH) signaling pathways are localized mainly in the glioma perivascular niche (Becher et al., 2008). Moreover, dysregulation of SHH signaling leads to enhanced proliferation of precursor cells likely to be involved in initiating the formation of gliomas (Clement et al., 2007; Komada, 2012). Moreover, the dysregulation of CXCL12/CXCR4 signaling induces aberrant astrocyte proliferation, which could also contribute to glioma progression (Bonavia et al., 2003). In response to CNS injury, reactive astrocytes can secrete IL‐6, tumor growth factor‐β (TGF‐β), insulin growth factor‐1 (IGF‐1), and tumor necrosis factor‐α (TNF‐α), (Seike et al., 2011; Placone et al., 2016). Furthermore, GDF-15 overexpressed in reactive astrocytes caused an enhanced proliferation of GBM cells *in vitro* while GDF-15 deficient cells exhibit decreased *in vivo* tumor growth (Roth et al., 2010; Zamanian et al., 2012). Astrocytes can also promote the invasion of CD133 + GBM stem Cells in the brain microenvironment (Rath et al., 2013). Overall, these processes directly or indirectly cause astrocytes transformation and promote gliomagenesis.

### Role of Cytokines

Cytokines are multifunctional molecules that control neo-angiogenesis, proliferation, invasion, and immune cell infiltration within the TME. Dysregulation of tumor-associated cytokine results in failure by the immune system to recognize tumor cells and thereby suppresses effective cell-mediated immunity (Zhu et al., 2012). Brain cells, tumor cells, and immune cells cross-talk through the complex cytokine network in the tumor microenvironment which promotes GBM progression (Iwami et al., 2011).

Glioma cells express multiple immune-suppressive cytokines such as transforming growth factor-beta (TGF-β), IL-10, IL-4, IL-6, and IL-13, all of which interfere directly or indirectly with anti-glioma immune response (Debinski et al., 1999; Gomez and Kruse, 2006; Perng and Lim, 2015; Brown et al., 2016; Harshyne et al., 2016). IL-33 has been shown to drive tumor progression, reduce overall survival, and orchestrate the GBM microenvironment to overcome resistance to immunotherapy (De Boeck et al., 2020). Both pro-inflammatory cytokines interleukin-6 (IL-6) and colony-stimulating factor-1 (CSF-1) trigger an immunosuppressive environment in GBM by inhibiting T-cell functions (Hao et al., 2002; Bender et al., 2010).

Despite the fact that most glioma-derived cytokines promote an inflammatory TME and are associated with glioma progression, some cytokines have been shown to have antitumor activity and have a therapeutic value. For example, IL-12 is endogenously secreted by antigen-presenting cells (APCs) which elicit adaptive cell-mediated immune responses (Heufler et al., 1996). However, despite the encouraging results of glioma eradication in mouse models, clinical studies using recombinant IL-12 in patients with advanced cancer were discontinued due to poor tolerability (Atkins et al., 1997; Leonard et al., 1997).

Another novel strategy used to target recurrent glioma is based on the differential expression of the IL-13 receptor by GBMs (IL13Rα2) that differs from the physiological IL4R/IL13R receptor expressed by normal cells. Adenovirus vector encoding mutated IL-13 (binds specifically to IL13Rα2) fused to active portion *Pseudomonas* exotoxin (Ad.mhIL-4. TRE.mhIL-13-PE) showed effective anti-GBM cytotoxicity, with minimal neurotoxicity (Kunwar, 2003; Hsieh and Lesniak, 2005; Mineharu et al., 2012). A similar strategy using a recombinant form of IL-13 fused to *Pseudomonas* Exotoxin A (IL13-PE38QQR) was introduced into GBM cells by convection-enhanced delivery (CED) method (Kunwar, 2003; Hsieh and Lesniak, 2005). This strategy is in multiple phase I/II clinical trials for the treatment of patients with recurrent malignant gliomas. Results from these trials indicated that local delivery of IL13-PE38QQR is safe and associated with an increase in median survival (Kunwar, 2003; Hsieh and Lesniak, 2005). Weiss et al. have recently developed fusion molecules that directly deliver cytokines to the tumors after systemic administration to promote immune responses. They combined immunostimulatory cytokines, such as IL2, IL12, or TNF, with an L19 antibody against GBM-specific fibronectin (Weiss et al., 2020). The resulting fusion immune-cytokines can be administered intravenously and accumulate in tumors, showing encouraging results in mouse models and a pilot study in human patients [NCT03779230]. Uncovering the modulatory role of glioma-derived suppressor factors and understanding the complex crosstalk between glioma cells and their microenvironment will assist in developing therapeutic strategies for glioma eradication.

### Role of Oxidative Stress

Chronic inflammation is the leading cause of oxidative stress in astrocytes and microglia.(Salazar-Ramiro et al., 2016). Excessive ROS production can induce damage at different cellular processes, such as metabolism, signaling molecules, and most importantly at the genomic level (including mitochondrial and chromosomal DNA).(Redza-Dutordoir and Averill-Bates, 2016). Changes in cellular ROS is one of the hallmarks that favor GBM cells’ survival and proliferation and provide further protection from apoptosis (Salazar-Ramiro et al., 2016). Genetic lesions commonly found in glioma are the leading cause of oxidative stress and genetic instability. GBMs exhibit epidermal growth factor receptor (EGFR) amplification, phosphatase and tensin homolog (PTEN) mutation, and loss of chromosome 10 all of which shift the balance toward higher ROS production within the tumor microenvironment (Reardon et al., 2015). For instance, constant activation of EGFR enhances the formation of intracellular ROS in neoplastic cells (Miller et al., 2007). This increase in ROS further enhances the tyrosine autophosphorylation by EGFR (Weng et al., 2018). Similarly, ROS can oxidize the PTEN protein and induce the disulfide bond formation between Cys71 and Cys124 within the N-terminal phosphatase domain resulting in the inactivation of the PTEN tumor suppressor activity (Lee et al., 2002; Kwon et al., 2004). Also, several studies suggested that TP53 mutation drives tumor progression by sustaining an oncogenic oxidant intracellular environment through altering the signaling pathways and of redox-related enzymes. For example, a mutation in TP53 deactivates mitochondrial-super oxide dismutase-2 (SOD2), and glutathione peroxidase (GPx), causing the accumulation of O_2_^−^ and H_2_O_2_, respectively (Hussain et al., 2004; Macedo et al., 2012; Cordani et al., 2020). All these processes induce aberration in the interstitial microenvironment and can drive glioma progression through excessive ROS production by tumor-infiltrating immune cells (Li et al., 2013). ROS-induced DNA damage triggers the release of a classical DAMP i.e. high-mobility group 1 (HMGB1) molecule which is released after oxidation of cysteine residues promoting genomic instability in GBM cells (Kang et al., 2013). TLRs can bind to the released HMGB1 and signal via the NF-κB, pathway causing enhanced production of pro-inflammatory cytokines, exacerbating ROS production in GBM cells [(Galdiero et al., 2013; Gieryng et al., 2017)].

The presence of ROS within the glioma TME can increase the immunosuppressive phenotype of immune cells infiltrating the TME, which interferes with effective immunotherapy. This immunosuppressive effect can be direct or indirect. Examples of direct ROS-mediated immunosuppression include the accumulation of Tregs (Kachikwu et al., 2011), M2-macrophages (Tsai et al., 2007), as well as interfering with dendritic cell activation and maturation (Kim et al., 2007). The indirect effect of ROS-mediated immunosuppressive TME includes the upregulation of PD-L1 by the tumor cells (Bailly, 2020; Raninga et al., 2020) as well enhanced expression of cytokines and chemokines that will recruit immunosuppressive myeloid cells (Bianchi et al., 2014; Eckert et al., 2018).

Studies also revealed that lower amount of nitric oxide (NO) produced through inducible nitric oxide synthase (iNOS/NOS2) in various tumors, including gliomas, can promote angiogenesis, invasion, and cellular proliferation (Fahey and Girotti, 2019; Maccallini et al., 2020). iNOS is a key regulator of glioma transformation downstream of the EGFRvIII/STAT3 signaling pathway. STAT3 directly binds to the promoter of the iNOS and thereby stimulates its expression (Jahani-Asl and Bonni, 2013). Moreover, iNOS-derived NO in glioma cells elicits resistance to various therapies including 5-aminolevulinic acid (ALA)-based photodynamic therapy (PDT) and endows gliomas of greater proliferation and aggressiveness (Fahey and Girotti, 2019; Maccallini et al., 2020). Several lines of evidence have showed that decreased proliferation of glioma cells could be mediated by selective inhibition of iNOS such as 1,400 W (N-(3-(aminomethyl) benzyl) acetamidine) (Fedorov et al., 2003) or mercapto-ethyl guanidine (MEG) (Southan et al., 1996) which inhibit the iNOS activity.

Generally, the CNS is composed of high fatty acids content which contributes to its high metabolic activity, making it particularly sensitive to oxidative damages by ROS (Panov et al., 2014). Neurons and astrocytes are equipped with antioxidant systems such as glutathione (GSSG-GSH) that defend these cells from oxidative damage. However, due to the high expression of SOD and catalase enzymes in astrocytes, there is an accelerated conversion of superoxide to hydrogen peroxide in these cells (Dokic et al., 2012). This makes astrocytes particularly sensitive to damage induced by ROS, leading to genetic instability and exacerbating neuroinflammation. The negative regulation of SOD-1 results in increased ROS production and enhanced phosphorylation of the signaling molecules Akt (Ghosh et al., 2010). This triggers rearrangement of the actin cytoskeleton downstream PI3K/Akt pathways; favoring the invasion and migration of GBM cells (Qian et al., 2005; Ghosh et al., 2010). Tumor cells exploit this inflammatory environment to invade, proliferate and sustain angiogenesis, by producing vascular endothelial growth factor (VEGF), Bv8, and MMP9 (Cao et al., 2013).

## Immunotherapy in Glioma

The concept of tumor immunotherapy involves activation of the immune system to eradicate the tumor by stimulating effector components or blocking immune suppressors. Glioblastoma is a “cold tumor” in that it harbors a low mutational load with low infiltrating cytotoxic T-cells, making it difficult to mount an effective immune response. Moreover, glioma is highly infiltrated by immunosuppressive immune cells such as regulatory T cells, and myeloid cells that actively promote inflammatory microenvironment and suppress the effector anti-tumor immune response. Despite all these challenges, Immunotherapy for glioma is a promising area of active investigation (Dunn-Pirio and Vlahovic, 2017; Kamran et al., 2018). Various preclinical studies have demonstrated the success of immunotherapy-based approaches in animal models and many phase I and II clinical trials have shown immunotherapy to be safe and in some cases lead to improved progression-free survival (PFS) (Phuphanich et al., 2013; Bloch et al., 2014; Mitchell et al., 2015). Below, we provide an overview of the immunological approaches which yielded promising results in the preclinical setting and are currently being tested in the clinic. A list of these trials are summarized in Table 1.

**TABLE 1:** Selected ongoing clinical trials of immunotherapy in glioma.

Target	NCT number	Drug	Disease	Status	References
Stat3	NCT01904123	WP1066	Recurrent GBM	Recruiting	Hussain et al. (2007)
Cox2	NCT00112502	Celecoxib	GBM	Completed	Penas-Prado et al., (2015), Mi et al. (2020)
Stat3	NCT04334863	WP1066	Brain metastases	Recruiting	Hussain et al. (2007)
Cxcr4	NCT01977677	AMD3100	Adult glioblastoma	Completed	Thomas et al. (2019)
avβ3 and avβ5 integrins	NCT00689221	Cilengitide	Glioblastoma	Completed	Russo and Cappoli, (2018), Roesch et al. (2018)
CSF1R	NCT01790503	PLX3397	GBM	Completed	Butowski et al. (2015), Colman et al. (2018)
CSF1R	NCT02829723	BLZ945, PDR001	Solid tumors	Recruiting	Butowski et al. (2015), Colman et al. (2018)
PD-1	NCT02017717	Nivolumab	Recurrent GBM	Active, not recruiting	Domingues et al. (2016), Woroniecka et al. (2018), Wang et al. (2019)
PD-1	NCT02550249	Nivolumab	GBM	Completed	Domingues et al. (2016), Woroniecka et al. (2018), Wang et al. (2019)
PD-1, CD8 T cells	NCT02529072	Nivolumab + DC vaccine	Astrocytoma	Completed	Peters et al. (2019)
TNF	NCT03779230	L19TNF	Glioma	Recruiting	Weiss et al. (2020)
IL-13Rα2	NCT02208362	Genetically modified T cells	Refractory malignant glioma	Recruiting	Kunwar. (2003), Hsieh and Lesniak, (2005)
EGFRv III	NCT01454596	CAR T cell	Malignant glioma	Completed	Migliorini et al. (2018)
HERs	NCT01109095	CAR T cell	GBM	Completed	Migliorini et al. (2018), Wang et al. (2020)
Tumor cells	NCT03576612	Ad-TK + valacyclovir	High-grade gliomas	Active, not recruiting	
Tumor cells	NCT02414165	Cytosine deaminase+ 5FU	GBM	Terminated	Cloughesy et al. (2020)
Antigen presenting cells	NCT02026271	Ad-RTS-hIL-12 + veledimex	GBM	Active, not recruiting	Chiocca et al. (2019), Garcia-Fabiani et al. (2020)
Antigen presenting cells	NCT03330197	Ad-RTS-hIL-12 + veledimex	Pediatric brain tumor	Recruiting	Chiocca et al. (2019), Garcia-Fabiani et al. (2020)
Antigen presenting cells	NCT04006119	Ad-RTS-hIL-12 + veledimex + PD1	Glioblastoma	Active, not recruiting	Chiocca et al. (2019), Garcia-Fabiani et al. (2020)
Dendritic cells	NCT01811992	Ad-hCMV-TK + Ad-hCMV-Flt3L	Malignant glioma	Active, not recruiting	Curtin et al. (2009), Mineharu et al. (2011), Lowenstein et al. (2019)
Dendritic cells	NCT00045968	Dendritic cell immunotherapy	GBM	Unknown	Vik-Mo et al. (2013), Hunn et al. (2015), Akasaki et al. (2016)
Dendritic cells	NCT00639639	Autologous dendritic cells	Brain tumor	Active, not recruiting	Batich et al. (2017)
Tumor-associated antigens	NCT04013672	SurVaxM (peptide vaccine)	Recurrent GBM	Active, not recruiting	Platten et al. (2018), Hollingsworth and Jansen, (2019)
Tumor-associated antigens	NCT03149003	DSP-7888 (peptide vaccine)	Glioblastoma	Active, not recruiting	Johanns and Dunn, (2017), Platten et al. (2018), Hollingsworth and Jansen, (2019)
Tumor-associated antigens	NCT02078648	SL-701 (peptide vaccine)	Adult GBM	Completed	Johanns and Dunn, (2017), Platten et al. (2018)
IDH1	NCT02454634	IDH1 peptide vaccine	Glioma	Completed	Platten et al. (2021)
Tumor cells	NCT02661282	Cytomegalovirus-specific cytotoxic T-lymphocytes	Glioblastoma	Active, not recruiting	Woo et al. (2012), Kim et al. (2017), Chheda et al. (2018)
CD200	NCT00648739	Samalizumab	Glioblastoma	Recruiting	Wright et al. (2000), Gorczynski et al. (2000), Wright et al. (2003)

### CAR-T Cells

Adoptive transfer of chimeric antigen receptor (CAR) T cells is a promising immunotherapy glioma treatment (Migliorini et al., 2018; Akhavan et al., 2019). CAR T cells are T cells that have been removed from the patients and modified to have tumor antigen-binding receptor specificity before adoptively transferring them back to the patient (Akhavan et al., 2019). This therapy increases the ability of T cells to recognize and target cancerous cells. The current CAR T cells in clinical trials target IL-13Rα2, epidermal growth factor receptor variant III (EGFRvIII), and human epidermal growth factor receptor 2 (HER2) (Migliorini et al., 2018). These targets were selected due to their expression by glioma cells and negligible expression by normal brain cells since CAR T cell targeting of normal brain cells could result in lethal levels of toxicity (Akhavan et al., 2019). The clinical trials, ClinicalTrials.gov NCT identifiers: NCT02208362, NCT01454596, NCT01109095, targeting IL-13Rα2, EGFRvIII, and HER2, respectively, provide useful insights on the immunotherapeutic ability of CAR T cells against gliomas (Migliorini et al., 2018). These trials showed the CAR T cells’: low toxicity, moderate ability to traffic to the glioma microenvironment, limited ability to target glioma cells, and inconsistent results in increasing overall survival (Migliorini et al., 2018; Akhavan et al., 2019; Wang et al., 2020). For example, HER2-specific CAR T cells, in the NCT01109095 trial, were used to treat 17 patients with progressive GBM (Ahmed et al., 2017). Of these, 8 patients had clinical benefits, and the overall survival of the cohort was 11.1 months post T cell infusion (Ahmed et al., 2017). Recently, several therapies have been designed to improve the efficacy of CAR T cells by combining them with checkpoint inhibitors, such as PD-1, CTLA4, and PD-L1, but the clinical trials are still ongoing and have not released results (Akhavan et al., 2019).

### HSC Transplantation

Hematopoietic stem-cell transplantation (HSCT) refers to a procedure in which hematopoietic stem cells are infused to restore bone marrow function in cancer patients who receive harmful doses of cytotoxic drugs or radiation therapy. Studies showed HSCs infusion is associated with enhanced recruitment and migration of tumor-specific T-cells within the tumor bed (Flores et al., 2015). This effect is mediated by HSCs-derived cytokines such as CCL3 (MIP-1α) that facilitates the subsequent recruitment of activated tumor-specific T cells causing enhanced anti-glioma immune response (Flores et al., 2015; Bryukhovetskiy et al., 2016). HSCs may be obtained from the transplant recipient (autologous HSCT) or a donor (allogeneic HSCT) and harvested from bone marrow, peripheral blood, or umbilical cord blood shortly after delivery of neonates. An early study compared survival outcomes of 27 children with recurrent malignant astrocytomas who underwent myeloablative chemotherapy and autologous HSCT with a matched historical cohort (n = 56) that received standard chemotherapy following tumor recurrence (Finlay et al., 2008). The results of this study suggest myeloablative chemotherapy with autologous HSCT can produce long-term survival among children with recurrent malignant astrocytoma. Bouffet et al. reported on a series of 22 children and young adults with high-grade gliomas treated with autologous HSCT, the response rate was 29% with one complete and three partial responses (Bouffet et al., 1997). However, the authors concluded that overall survival using this procedure was no better than that reported with conventional treatments (Bouffet et al., 1997). . In conclusion, HSC therapy may extend glioma patients' survival particularly for those patients being treated with high dose chemotherapy regimens. However, the dose of chemotherapeutics used in this treatment regimen should be carefully balanced to avoid intolerance and toxic side effects (Brandes et al., 2001; Egan et al., 2016).

### Gene Therapies and Virotherapies

Gene therapy is a therapeutic approach that involves the insertion of the genetic material into a tissue or organ to treat a variety of diseases, including solid cancers, such as brain tumors. These genetic elements include whole genes, oligonucleotides, or regulatory elements which are usually inserted into the target cells either by gene delivery vectors or liposomal-nanoparticle formulations. (Asad et al., 2017; Kamran et al., 2018). In glioma, gene therapy is an attractive approach due to the fact that its local administration can overcome the challenges needed to bypass the BBB, and therefore it may have lower systemic side effects (Okura et al., 2014). Albeit, its clinical application still presents many challenges (Lowenstein et al., 2009).

Among prevalent gene therapy approaches for cancer, suicide gene therapy is one of the most studied approaches for the treatment of glioma. Currently, there are around 20 Phase-I/II clinical trials testing the effectiveness of adenoviral vectors mediated suicide gene therapies in multiple subtypes of glioma. An ongoing Phase-I trial evaluates the safety and tolerability of Ad-TK in combination with systemic administration of the prodrug valacyclovir in combination with SOC and Nivolumab in patients with HGG (NCT03576612). The trial is still ongoing with no results posted.

Another example of a clinically applied conditional cytotoxic/suicidal approach is the delivery of the cytosine deaminase (CD) in cancer cells. CD is expressed mainly by yeast and bacteria but is absent in mammalian cells (Takahashi et al., 2014). GBM cells expressing CD will be susceptible to 5-fluorocytosine, due to it is the ability to convert it to its active form Fluorouracil (5-FU) (Okura et al., 2014; Takahashi et al., 2014). The initial Phase-I clinical trial of Toca 511, a replication-competent RV encoding CD, showed safety and good tolerability, with effective tumoricidal responses in patients with recurrent high-grade glioma (Cloughesy et al., 2018). However, a recent phase III clinical trial (NCT02414165) did not show improved overall survival compared to the SOC (Cloughesy et al., 2020).

Another attractive approach is immune stimulatory gene therapy which entails the expression of immune activators in glioma cells to enhance the anti-tumor immune response.

Two independent Phase-I trials (NCT02026271, NCT03330197) investigating the tolerability of Ad-RTS-hIL-12 in combination with veledimex (VDX) showed promising results (Chiocca et al., 2019). In a separate Phase-I trial, the safety of Ad-RTS-Hil-12/VDX is being tested in combination with Nivolumab (NCT03636477) (Garcia-Fabiani et al., 2020). In addition, a Phase-II trial is studying the inducible hIL-12 in combination with PD-1 antibody Libtayo (NCT04006119) (Garcia-Fabiani et al., 2020).

In an effort to overcome the shortcomings of monotherapies, combination therapies have been developed. We have pioneered the combination of Ad-Flt3L and Ad-TK (Castro et al., 2014; Kamran et al., 2018). In this therapeutic approach, adenoviral vectors encoding for herpes simplex type 1-thymidine kinase (TK) and FMS-like Tyrosine kinase 3 ligand (Flt3L) are delivered into the brain where glioma cell death is induced upon systemic ganciclovir treatment (Ali et al., 2005). This triggers the release of tumor-associated antigens and damage-associated molecular pattern molecules (DAMPs) into the tumor microenvironment, triggering an antitumor immune response (Kamran et al., 2018; Altshuler et al., 2020). Our results indicate that the release of DAMPs such as HMGB1 from Ad-TK infected tumor cells is required for the efficacy of Ad-TK + Ad-Flt3L mediated immunotherapy (Candolfi et al., 2009; Curtin et al., 2009). Flt3L increases the migration and infiltration of DCs into the TME. This glioma infiltrating DCs can phagocytose antigens that are released during TK-induced glioma cell death (Curtin et al., 2009; Candolfi et al., 2012). Moreover, HMGB1 released from dying tumor cells activates DCs through stimulation of TLR2 pathway. These activated DCs then transport the tumor antigens to the draining lymph nodes to generate T-cell mediated cytotoxic immune responses against tumor cells (Curtin et al., 2009). This strategy effectively eradicated the tumor in multiple glioma models and granted long-term survival in tumor-bearing mice (Mineharu et al., 2011). Results from Phase-I dose-escalating trial (NCT01811992) showed well-tolerability and safety when giving a combination of AdVs expressing TK and Flt3L. There was also higher infiltrating DCs and lymphoid cells within the TME patients receiving the gene therapy (Lowenstein et al., 2019).

### Dendritic Cells Vaccines

Dendritic Cells (DCs) are professional antigen-presenting cells (APC) that are able to engulf antigenic proteins, process peptides, and traffic to the draining lymph nodes (dLNs) where they present antigens to naive T-cells (Palucka and Banchereau, 1999). This process leads to induction and activation of effector T-cell, the goal standard for anti-tumor immune response. Administration tumor antigen pulsed-DCs has been validated in multiple solid tumors such as melanoma, prostate cancer, and renal cell carcinoma (Schadendorf et al., 2006; Su et al., 2003; Anguille et al., 2014). In glioma, DCs therapy represents an attractive approach especially because of the paucity of professional DCs in GBM (Eagles et al., 2018; Srivastava et al., 2019). Preclinical models have shown promising results for DCs treatment in combination with PD-1 blockade (Antonios et al., 2016). In addition, phase I clinical trial revealed that the combination of TMZ with DCs therapy is safe and tolerable in GBM patients (Vik-Mo et al., 2013; Hunn et al., 2015; Akasaki et al., 2016). Moreover, autologous DCs pulsed with autologous tumor lysate (DCVax) are currently being investigated in phase III clinical trials for newly diagnosed GBM (NCT00045968). Beside the direct administration of autologous DCs to glioma patients, the activation and recruitment of endogenous DCs *in vivo* have been shown to be safe and effective in glioma patients. This is illustrated by infusion of TLR ligands, or by intratumoral injection of Flt3L which causes robust DCs expansion and activation (Hou et al., 2008; Belderbos et al., 2019; Jones et al., 2010)*.* Another trial tests the efficacy of DCs loaded with glioma-associated protein (pp65) along with the lysosomal associated protein (LAMP) (CMV-pp65-LAMP mRNA-loaded DCs) in combination with the TMZ and GM-CSF. This study showed increased progression-free survival (PFS) and OS, and upregulation in IFNγ levels (Batich et al., 2017) (NCT00639639). These trials showed that DCs therapy is safe, and well-tolerated in glioma patients and elicited a minimal inflammatory response. Future trials will further reveal the effectiveness of this treatment on patients with recurrent GBM and its impact on overall survival.

### Peptide Vaccines

Peptide vaccines are developed based on peptides derived from tumor-associated antigens (TAAgs) and used to induce effective anti-tumor T-cell response (Lee et al., 2020). Some of the TAAgs tested in glioma, survivin, WT-1 protein, EphrinA2, and IL13RA2, have been investigated as potential peptide vaccines (NCT04013672, NCT03149003, NCT02078648); however, therapeutic efficacy has been limited (Platten et al., 2018). The low efficacy of TAAgs vaccines could be attributed to the tolerance mechanisms to self-antigens, which also implies the risks for off-target toxicity (Hollingsworth and Jansen, 2019). Alternatively, neoantigens that are exclusively expressed in tumor cells can be employed in peptide vaccines. The major challenge for glioma lies in the epitope selection: it harbors only 30–50 non-synonymous mutations per megabase in glioblastomas, and roughly 1–3% of the mutations result in immunogenic neoantigens (Schumacher and Schreiber, 2015; Johanns and Dunn, 2017). Despite the low mutational burden, some shared neoepitopes, such as EGFRvIII and IDH1R132H, are identified, and corresponding vaccines have been extensively evaluated in clinical trials (Weller et al., 2017; Platten et al., 2018; Reardon et al., 2020). Recently, a peptide vaccine targetting the most common mutation in glioma *IDH1*m (R132H) has been developed and showed promising results in Phase-I clinical trial (NCT02454634) (Platten et al., 2021). Overall, neoantigen vaccines demonstrate good safety and immunogenicity, but the response rate and survival benefits are marginal. It has been recognized that neoantigens are usually unique, and the subclonal expression would lead to spontaneous antigen loss, opening avenues for immune escape and tumor progression (Lim et al., 2018).

### High-Density Lipoprotein Nanoparticle Vaccines

The tantalizing results and heterogeneity of neoantigen expression emphasize the need for the development of personalized vaccination. Currently, neoantigen identification is based on the whole-exome sequencing (WES), followed by computational predications of the antigenicity through HLA binding (Kreiter et al., 2015), which can be further confirmed by the transcriptome analysis with mass spectrometry (Yadav et al., 2014). In particular, Kuai, et al. have developed a sHDL nanodisc composed of phospholipids and apolipoprotein A1-mimetic peptides for neoantigen vaccination (Kuai et al., 2017). After coupling with Cytosine-phosphorothioate-guanine CpG oligonucleotide, a TLR-9 agonist, nanodiscs promoted co-delivery of Ag/CpG to lymphoid organs, eliciting strong neoantigen-specific anti-tumor CD8^+^ T-cell responses in murine models of melanoma and colon carcinoma. Taking one step further, Scheetz et al. developed the personalized peptide vaccine against gliomas with the sHDL nanodisc platform (Scheetz et al., 2020). Three neoantigens of GL261 tumor cells were screened by *in silico* MHC-I affinity prediction algorithms and subsequent immunogenicity verification in tumor-bearing mice. Selected peptides were loaded to nanodiscs together with CpG. Immunizing mice with the vaccine plus anti-PD-L1 therapy improved the survival rate to approximately 90% in the subcutaneous GL261 tumor model, and it showed 33% complete tumor regression in the orthotopic model, which represented a significant survival advantage compared with soluble neoantigens plus anti-PD-L1 treatments. Nanodisc vaccination elicited robust IFN-γ^+^ T cell response against all three epitopes, and the addition of anti-PD-L1 further augmented the T cell responses. TME analyses on CNS tumors showed an increased frequency of CD8^+^ T cells with lower levels of PD-1 expression. Meanwhile, compared with the PBS group, lower Tregs frequency and higher M1:M2-like macrophage ratio was observed in tumor tissues, suggesting a reversed immunosuppressive milieu. In parallel, the therapeutic efficacy of nanodiscs with either mIDH1_123–132_ or mIDH1_126–141_ neoantigens was evaluated in a genetically engineered mIDH1 murine glioma model. The results showed that nanodiscs vaccination significantly suppressed tumor growth and extended the survival rate. All survived mice showed resistance to tumor recurrence upon rechallenge with mIDH1 neurospheres in the contralateral hemisphere.

Aside from TAAs and neoantigens, cancer stem cells (CSCs) and other non-neoplastic compartments may serve as potential targets for gliomas. Nanodiscs delivering CSC-derived ALDH1-A1 and -A3 peptides have been shown to induce potent anti-tumor activity and prolong animal survival in multiple mouse models (Hassani Najafabadi et al., 2020). Overall, sHDL nanodisc serves as a versatile platform with potent anti-tumor efficacy.

### Other Immunotherapies

Immune checkpoint inhibitors targeting the program death receptors and its ligand (PD-1/PD-L1) and T lymphocyte-associated antigen-4 (CTLA-4) have significantly enhanced immunotherapy efficacy for selected solid tumors (Ferris et al., 2010; Ferris et al., 2016; Bouffet et al., 2016). However, CNS tumors at best have a moderate response to current immunotherapies (Kurtulus et al., 2015; Bouffet et al., 2016). Recently, there has been increasing interest in alternative immunotherapy strategies including the synergy between PD1, TIM3, and LAG3 blockade and CAR T cells therapy (NCT02661282) for the treatment of gliomas [(Woo et al., 2012; Ahmed et al., 2017; Kim et al., 2017; Chheda et al., 2018)]. Recently, there have been advances with an alternative immune checkpoint using a novel immune checkpoint inhibitor peptide ligand. The CD200 immune checkpoint is modulated by an inhibitory receptor (CD200R1) and multiple activation receptors (CD200AR) (Wright et al., 2000; Gorczynski et al., 2000; Wright et al., 2003; Gorczynski et al., 2004; Wong et al., 2012; Damiani et al., 2015). Rigorous studies by several groups have provided evidence that targeting the CD200 checkpoint enhances immunotherapy efficacy (Kretz-Rommel et al., 2007; Copland et al., 2007; Gorczynski et al., 2011; Rygiel et al., 2012; Gorczynski et al., 2013). To this end, a monoclonal antibody against the CD200 protein (Samalizumab) (Adler, 2010) was evaluated in a clinical trial in 2008 for relapsed refractory B-cell chronic lymphocytic leukemia (B-CLL) and multiple myeloma (NCT00648739) (Adler, 2010). Alexion reported that 95% of the patients with B-CLL had up to 98% reduction in CD200^+^CD4^+^ T cells. The loss of these immune cells created an immunocompromized state and may be the reason why only 36% of the patients had a 10% reduction in bulk disease. Investigators have taken a new approach for improving immunotherapies by targeting the CD200ARs on antigen-presenting cells with a peptide ligand. They reported that the peptide ligand activates the immune system enhancing cytokine/chemokine production, Co-stimulatory molecules CD80/CD86 and MHC-II enhancing an antigen response while downregulating the inhibitory CD200R1 and PD-1 (Moertel et al., 2014; Xiong et al., 2016; Xiong et al., 2019). They reported that treatment with autologous tumor lysate given concomitantly with the peptide ligand significantly enhanced survival in a spontaneous canine high-grade glioma trial with pet-owned dogs (Olin et al., 2019) and recently initiated a phase I adult glioblastoma recurrent trial (NCT04642937) (Table 1).

## Prospects and Conclusion

In this review, we discuss multiple mechanisms by which gliomas induce the development of an immunosuppressive TME and evade immune surveillance. Various components within the tumor microenvironment such as secreted immune-modulating factors, hypoxia, and oxidative stress result in activation of the surrounding astrocytes and microglia to an inflammatory state. This suppressive microenvironment is further enhanced by infiltrating immunosuppressive myeloid cells, immunosuppressive macrophages, exhausted T cells, and Tregs which contribute to a tumor-supportive TME, promoting glioma cell growth and invasion. We also discuss recent advances in immunotherapeutic strategies that if used in combination could provide promising novel approaches that could elicit clinical benefit. These include adding immune checkpoint blockade to immune-mediated gene therapies, virotherapy, CAR-T cells, dendritic cells’ vaccines, or nanoparticle-mediated vaccination technologies.

Despite the advances in treatment regimen in multiple solid tumors in recent years, the SOC for glioma has not changed since 2005 with the inclusion of TMZ. The prognosis for GBM remains dismal with no major improvements in the median or overall survival. Major challenges need to be overcome in gliomas, such as tumor heterogeneity, the immunosuppressive TME (MDSCs, TAMS, Tregs), the low mutational burden, and the antigen heterogeneity in order to elicit effective immunotherapies. Further studies are crucial to delineate the complex mechanisms and interactions between tumor cells, immune cells, tumor stroma, resident brain cells, and the brain and tumor vasculature.

Over the past 2 decades, a pressing need to deeply profile the glioma TME has led investigators to integrate data obtained from traditional approaches with those obtained with new, single-cell technologies, including high parameter mass cytometry, single-cell sequencing, and high-resolution imaging. These advances have enabled us to investigate in detail the complexity of the TME and to interrogate in-depth previously unexplored tumor-infiltrating cell types. This expanded our understanding of the multifaceted tumor ecosystems and allowed us to profile cellular heterogeneity, dynamicity and plasticity, and complex cell-cell interactions.

Current open clinical trials testing immunotherapy in GBM could provide promising avenues for gene therapy, CAR-T cells as well as vaccine therapies in various combinatorial treatment strategies. These combinatorial approaches which have proven beneficial in the preclinical setting could prove particularly valuable in the clinical arena, when targeting multiple immune checkpoints or when adding cytokine therapy, likely to result in better outcomes. Furthermore, recent results from trials of neoadjuvant immune checkpoint blockade provide a new cause for optimism as we work to decode mechanisms for treatment resistance. On the other hand, an increased understanding of the possible indicators of side effects of immunotherapies will lead to improved clinical care and make the application of immunotherapies far safer and well-tolerated for glioma patients. Despite formidable challenges, with the advent of single-cell transcriptomic analysis (Wang et al., 2019; Gibellini et al., 2020; Mathewson et al., 2021), high parameter mass cytometry (Hartmann et al., 2019; Wang et al., 2019; Gohil et al., 2021), and high-resolution Hyperion imaging technologies (Carvajal-Hausdorf et al., 2019; Xie et al., 2020), the glioma immunotherapy field is undergoing unprecedented growth in the discovery of novel therapeutic targets and interventions, that we envisage will lead to effective novel treatments for this devastating brain cancer in the near future.
